# Occurrence and genetic characterisation of *Acanthamoeba* spp. from environmental and domestic water sources in Queen Elizabeth Protected Area, Uganda

**DOI:** 10.1186/s13071-016-1411-y

**Published:** 2016-03-03

**Authors:** Celsus Sente, Joseph Erume, Irene Naigaga, Phillip Kimuda Magambo, Sylvester Ochwo, Julius Mulindwa, Benigna Gabriella Namara, Charles Drago Kato, George Sebyatika, Kevin Muwonge, Michael Ocaido

**Affiliations:** Department of Wildlife and Aquatic Animal Resources (WAAR), School of Veterinary Medicine and Animal Resources (SVAR), College of Veterinary Medicine, Animal Resources and Biosecurity (COVAB), Makerere University, P.O. Box 7062, Kampala, Uganda; Department of Biomolecular Resources and Biolab Sciences, School of Biosecurity, Biotechnical and Laboratory Sciences (SBLS), College of Veterinary Medicine, Animal Resources and Biosecurity (COVAB), Makerere University, P.O. Box 7062, Kampala, Uganda; Medical Research Council (MRC)/Uganda Virus Research Institute (UVRI), Research Unit on AIDS, P.O. Box 49, Entebbe, Uganda; Department of Biochemistry and Sports Science, College of Natural Sciences (CONAS), Kampala, Uganda

**Keywords:** *Acanthamoeba*, Domestic, Environment, Water, Uganda

## Abstract

**Background:**

*Acanthamoeba* is an emerging potentially pathogenic amoeba that has been receiving increasing attention worldwide as a reservoir and potential vector for the transmission of pathogenic bacteria. It is also associated with brain cell damage, keratitis and skin irritation in humans. Its effects are more severe in immunocompromised individuals. This study provides for the first time in Uganda, information on the prevalence and genotypes of *Acanthamoeba* in environmental and domestic (tap) water.

**Methods:**

A total of 324 environmental and 84 tap water samples were collected between November 2013 and September 2014. The samples were centrifuged, cultured (Non-Nutrient agar seeded with gram-negative bacteria) and observed under a microscope. After confirmation of *Acanthamoeba*, genomic DNA was extracted for PCR assays by chemical lysis and purification with phenol/chloroform/isoamyl alcohol. Samples that showed the strongest positive bands (400–600 bp) were subjected to cycle sequencing.

**Results:**

Among environmental and tap water samples, 107 (33 %) and 36 (42.9 %) tested positive for *Acanthamoeba* spp., respectively. Prevalence of *Acanthamoeba* from specific environmental locations was as follows; Kazinga channel banks (60.7 %), Fish landing sites (50 %), River Kyambura (39.6 %) and Kazinga mid channel (5.3 %). There was a significant difference (*p* = 0.001) in the prevalence of *Acanthamoeba* between sampling sites. The mean (Mean ± SE) occurrence of the organism was higher in Kazinga channel banks (3.44 ± 0.49) and Fish landing sites (3.08 ± 0.53). Correlation between in situ parameters and *Acanthamoeba* was insignificant except for the Dissolved Oxygen (mg/ML) which was negatively correlated (*r* = −0.231, *p* = 0.001) to *Acanthamoeba.* Six distinct partial *Acanthamoeba* T-genotype groups T1, T2, T4, T5, T6 and T11 were obtained. Ultimately, *Acanthamoeba* spp., *Acanthamoeba hatchetti* and *Acanthamoeba polyphaga* were isolated in the current study.

**Conclusions:**

There was a high prevalence of *Acanthamoeba* in communal piped tap and environmental water used by communities, indicating poor environmental and domestic water quality.

## Background

Rural communities in and around Queen Elizabeth Protected Area (QEPA) are directly or indirectly dependent on environmental sources of water (fresh water lakes, rivers, streams, water holes, and gravity water) for all their water needs including; drinking, washing, bathing, recreation and agriculture. A significant portion of these communities using environmental or piped tap water sources could be exposed to the risk of contracting waterborne diseases. Waterborne diseases such as cholera, campylobacteriosis, shigellosis, salmonellosis and typhoid among others, are known to affect proportions of rural communities [1, 2]. Several other waterborne diseases are not often mentioned and yet they might have potential pathogenic effects. Disease effects associated with free-living amoeba (FLA) in humans and animals in Uganda often go undetected because little is known about their presence and distribution in certain environments.

Water and soil-dwelling amoebae are widespread in nature and have been isolated from a variety of engineered water systems, aquatic and terrestrial environments [3–5]. Some FLA of the genera *Acanthamoeba*, *Balamuthia*, *Naegleria* and *Hartmannella* occasionally invade hosts and cause infections [3, 6]. *Acanthamoeba* is both opportunistic and pathogenic with two stages in the life-cycle, an active trophozoite that exhibits vegetative growth and a dormant cyst stage with minimal metabolic activity [3, 7]. *Acanthamoeba* renders possible intracellular multiplication of *Bacillus anthracis*, *Legionella pneumophila*, *Vibrio cholerae* and *Mycobacterium tuberculosis* which are responsible for anthrax, legionellosis, cholera and tuberculosis respectively [7–9]. Certain *Acanthamoebae* spp. are also causative agents of granulomatous amoebic encephalitis (GAE), a fatal disease of the CNS, amoebic keratitis (AK), a painful sight-threatening disease of the eyes [8, 9] and have also been associated with cutaneous lesions with sinusitis in HIV/AIDS patients and other immunocompromised individuals [8, 10].

Although *Acanthamoeba* spp. are causative agents of various diseases and carriers of pathogenic bacteria, little is known about their occurrence in Uganda. We, therefore, report for the first time in Uganda the occurrence and genotypic classes of *Acanthamoeba*.

## Methods

### Study area

The study was conducted in the Queen Elizabeth Protected Area (QEPA) that spans the districts of Rubirizi, Kasese, Rukungiri and Kamwenge in Uganda. The QEPA is a 1,978 sq. km with coordinates 00 12S, 30 00E (Latitude: 0.2000; Longitude: 30.00 C00). The vegetation cover is savannah grassland mixed with forest, woodland and wetland ecosystem. The protected area harbours Lake George and Edward that are joined by a 40 km long Kazinga channel. Within the protected area are 11 fishing enclaves, with a population of mostly fishermen and pastoral communities.

### Ethics

This study does not require ethical approval.

### Sample collection, storage and transportation

The locations for sample collection were purposively selected based on certain landmarks such as nearness to burial sites/mass graves of hippos that died of anthrax (water sources adjacent to the graves) and nearness to the fishing villages where communities fetch water for domestic use. The water sources included environmental water and communal piped tap water. Environmental water sites were derived as collection points on (1) River Kyambura (R. Kyambura), (2) Kazinga channel bank (KCB), (3) Fish landing site (FLS) and (4) Kazinga mid channel (KMC). The fifth site (communal piped tap water system) is fed by water from the environment (Kazinga channel and River Kyambura) after it is treated.

Important in situ water parameters including; dissolved oxygen (mg/l), surface water temperature (°C), electrical conductivity (μS/m), pH, total dissolved solids (g/l) and oxidation reduction potential (mV) were determined on-site using a Multi-parameter water sensor (Greenspan, USA). Four hundred and eight water samples were collected using 50 ml sterile polypropylene falcon tubes (Discovery Labware, USA). A total number of 408 (324 environmental and 84 tap) water samples were collected. The samples were stored at room temperature and transported to the Makerere University Molecular Biology Laboratory (MOBILA) within 48 h for culturing and isolation of *Acanthamoeba.*

### Laboratory analysis

This was done by culturing the collected water samples for *Acanthamoeba* spp. and examining for the presence of *Acanthamoeba* trophozoites under the microscope, followed by DNA extraction, DNA amplification and sequencing.

#### Amoeba cultivation (Non – Nutrient agar seeded with gram-negative bacteria)

The NN-EI non-nutritive medium (Page Amoeba Saline solution-2.5 mM NaCl, 1 mM KH_2_PO_4_, 0.5 mM Na_2_HPO_4_, 40 mM CaCl_2_, and 20 mM MgSO_4_) seeded with 0.1 ml of a heat inactivated 48-h culture of *Escherichia coli BL21* was prepared and the final pH of the solution adjusted to 6.9. Water in the 50 ml tubes was centrifuged at 1000 × *g* for 15 min and the supernatant poured off to expose the pellet which was spread on the already prepared agar plates. The plates were incubated at 30–32 °C overnight. After one day, the plates were wrapped in polyethene bags and incubated upside down at 30–32 °C up to 7 days. After 3 days of incubation, the plates were monitored for detection of *Acanthamoeba* trophozoites microscopically daily until the 7^th^ day. The number of *Acanthamoeba* trophozoites were counted using haemocytometer (MicrobeHunter, Germany) and recorded.

#### Identification of Acanthamoeba at genus level

In order to determine the genus of the protozoa, movement and structural properties of amoebae were examined. Distinguishing features of the *Acanthamoeba* trophozoites were the presence of spiny or fingerlike surface projections called *acanthopodia*, a prominent contractile vacuole in the cytoplasm and vesicular nucleus with large central nucleolus [11].

### Molecular identification of the isolates and phylogenetic analysis

#### Reference strains

The reference amoeba strain used in this study was the Amplirun® *Acanthamoeba castellanii* DNA Control, MBC054, and was the positive control for in vitro diagnosis techniques based on nucleic acids amplification (Labconsult, Bruxelles-Brussels, Belgium).

#### Polymerase chain reaction (PCR) amplification assay for acanthamoebae

After identifying amoebae from the water samples, genomic DNA was extracted for PCR analysis by chemical lysis and purification of DNA with phenol/chloroform/isoamyl alcohol extraction method [12]. Five hundred microliters of STE buffer (0.1 M NaCl, 1 mM EDTA, 10 mM Trischloride, PH 8, 1 % SDS) and 10 μl proteinase K (10 mg/ml) was added directly to each sample in an Eppendorf tube. The samples were incubated at 56 °C for one hour and then cooled before phenol extraction. An equal volume of phenol-chloroform (521 μl) was added to each sample, mixed by vortexing and centrifuged at 13,200 rpm for 10 min. The aqueous layer was recovered and transferred to a new Eppendorf tube. This step was repeated to make two phenol-chloroform extractions. The aqueous layer from each tube was subjected to another chloroform extraction, recovered by centrifugation and transferred to a new Eppendorf tube after which 1000 μl of absolute alcohol (96–100 %) was added. The samples were then put in a freezer at −80 °C for precipitation overnight. The next day, samples were removed from the freezer and centrifuged at 13,200 rpm for 30 min. After 30 min the absolute alcohol was poured off. The pellet in each tube was then washed with 1000 μl of 70 % alcohol, centrifuged at 13,200 rpm for 15 min and alcohol poured off to expose the pellet. Finally, the pellet was air dried and dissolved in 50 μl of TE buffer.

Amplification of the partial 18S ribosomal RNA (18S rRNA) gene from *Acanthamoeba* was performed using primers previously shown to be specific for *Acanthamoeba* spp. [13, 14]. The primer pairs were: forward primer JDP1 (5’GGCCCAGATCGTTTACCGTGAA-3’) and reverse primer JDP2 (5’TCTCACAAGCTGCTAGGGAGTCA-3’).

Amplification reactions were performed using a DreamTaq PCR kit (Thermoscientific DreamTaq, USA). We used a 25 μl reaction volume containing 12.5 μl DreamTaq Green PCR Master Mix (2X), 0.5 μM forward primer, 0.5 μM reverse primer, 9 μl nuclease free water and 2.5 μl DNA template (50 pg concentration). The PCR was done under the following conditions: Initial denaturation at 94 °C for 3 min then 35 cycles with denaturation at 94 °C for 30 s, annealing at 55 °C for 30 s, extension at 72 °C for 30 s and final extension at 72 °C for 5 min. A sample of 5 μl of each PCR reaction was screened for successful amplification on a 2.5 % (W/V) agarose gel stained with ethidium bromide and run against 1kbp DNA ladder (Finnzymes, Finland). Electrophoresis was performed at 100 V of current and buffer used was 1X TAE containing 0.5 μg/ml of ethidium bromide. Once enough electrophoretic separation was obtained, the agarose gel was visualised using a UV gel documentation system (Wagtec, UK). The gel images were captured and a soft copy stored.

#### Nucleic acid sequencing and analysis

Samples that showed the strongest positive bands (400–600 bp) were extracted from the gel and the DNA was purified using QIAquick gel extraction kit (Qiagen Inc. Sample and Assay Technologies, Netherlands). The 18S rDNA segment from each of the *Acanthamoeba* isolates was subjected to cycle sequencing by the dideoxynucleotide chain termination method using the Dyenamic Terminator Cycle sequencing kit with JDP as sequencing primer [14]. The sequencing included 2 μl of PCR product, 5ХBigDye Buffer, and 2pmol primer. Sequencing was done in 30 cycles with step 1 at 94 °C for 30 s, step 2 at 55 °C for 15 s and step 3 at 65 °C for 4 min.

The sequence files were checked for quality and base trimming carried using the Seqbuilder software (Dnastar, USA). For each of the nucleotide query sequences, a search for homologues in the NCBI database was carried out using the blastn tool. Homologues with query coverage > 80 %, identity > 70 % and low E values (<0) were considered. The molecular phylogenetic analysis was then carried out using the Maximum Likelihood Method in MEGA6 [15].

### Statistical analysis

Data was analysed using IBM SPSS version 22. Numerical variables were summarised using mean and standard error of the mean (SEM). Univariate analysis to compare the prevalence of *Acanthamoeba* across sampling sites was done using cross-tabulation with a Chi-square or Fisher’s exact test. Variables with a *p*-value of ≤ 0.05 were taken to be significant. Correlation analysis between environmental variables and *Acanthamoeba* presence was done using Pearson correlation coefficient (r), a *p*-value of ≤ 0.05 was considered statistically significant.

## Results

### Prevalence of *Acanthamoeba* per water site source

*Acanthamoeba* prevalence in environmental and tap water samples was 33 % and 42.9 % respectively (Table 1). The prevalence in various environmental sites where samples were collected was as follows: River Kyambura (39.6 %), KCB (60.7 %), FLS (50 %) and KMC (5.3 %) (Table 2). The results show that the number of organisms isolated was significantly (*p* = 0.001) influenced by the sampling site.

**Table 1:** Prevalence of *Acanthamoeba* by water source

	*Acanthamoeba*		
Source	Number of samples	Number positive	Prevalence (%)
Tap Water	84	36	42.9
Environmental Water	324	107	33
Total	408	143	65

**Table 2:** Presents prevalence, means and in situ parameters per sampling site

			*Acanthamoeba*			In situ parameters				
					DO(mg/l)	Temp(°C)	Cond(μS/m)	pH	TDS(g/l)	ORP(mV)
Site	No.	+ve	Prev (%)	Mean(±SE)	Mean(±SE)	Mean(±SE)	Mean(±SE)	Mean(±SE)	Mean(±SE)	Mean(±SE)
R. Kyambura	48	19	39.6	2.23 ± 0.53	4.03 ± 0.16	21.43 ± 0.15	150.98 ± 3.02	7.99 ± 0.11	113.12 ± 6.42	−541.9 ± 52.99
Kazinga channel bank	84	51	60.7	3.44 ± 0.49	1.74 ± 0.15	25.63 ± 0.24	273.71 ± 9.27	9.31 ± 0.04	182.76 ± 4.87	−467.8 ± 29.30
Fish landing sites	60	30	50	3.08 ± 0.53	1.84 ± 0.21	26.22 ± 0.34	280.99 ± 10.36	8.91 ± 0.12	183.38 ± 6.84	−412.5 ± 33.97
Kazinga mid channel	132	7	5.3	0.17 ± 0.08	4.57 ± 0.26	25.76 ± 0.20	261.75 ± 6.78	9.18 ± 0.07	163.09 ± 4.04	−454.2 ± 25.09
Tap water	84	36	42.9	2.26 ± 0.4	4.08 ± 0.27	26.89 ± 0.36	163.26 ± 111.69	7.73 ± 0.10	108.94 ± 7.50	−322.8 ± 23.25

The means (Mean ± SEM) of the organism in water across sites were as follows from highest to lowest; KCB (3.44 ± 0.49), FLS (3.08 ± 0.5), Tap water (2.26 ± 0.4), R. Kyambura (2.23 ± 0.53) and KMC (0.17 ± 0.08). The highest *Acanthamoeba* prevalence and means (KCB and FLS) were associated with lower DO (mg/l), higher, Temperature (°C), conductance (μS/m) and TDS (g/l) (Table 2).

### Correlation of *Acanthamoeba* presence and in situ parameters

Dissolved oxygen (DO) was negatively correlated (*r* = −0.231^**^) with *Acanthamoeba* spp. There was a weak negative correlation (*r* = −0.051) between water temperature and *Acanthamoeba* spp. The other parameters (Conductance, pH, TDS and ORP) were positively correlated to the *Acanthamoeba* spp. (Table 3).

**Table 3:** Correlation coefficient (*r*) between environmental variables and *Acanthamoeba* presence

*In situ* parameter	Correlation coefficient (*r*)	Significance
DO (mg/ML)	−.231^**^	0.001
Temp (°C)	−.051	0.3
Cond (μS/m)	.090	0.07
pH	0.5	0.31
TDS(g/L)	0.09	0.09
ORP(mV)	0.2	0.059

**very significant

### Molecular identification of the isolates and phylogenetic analysis

**Fig. 1:**
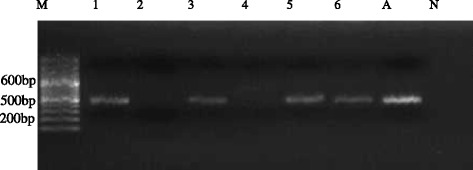
Agarose electrophoresis (2.5 %) showing amplification of JDP-PCR of *Acanthamoeba*. Lane M = DNA Ladder (100 bp), Lane A = Positive control, Lane N = Negative control, Lanes 1, 3, 5 & 6 = *Acanthamoeba* positive PCR product from obtained water samples

Amplicon sizes between 400 and 600 bp were observed (Fig. 1). Six distinct partial *Acanthamoeba* sequences belonging to the group of sequence types, T1, T2, T4, T5, T6 and T11 were obtained. The sequences were 98–99 % similar; with only two identical to already known *Acanthamoeba* sequences. Four sequences were not identical to known *Acanthamoeba* in NCBI. Ultimately, *Acanthamoeba* spp., *Acanthamoeba hatchetti*, and *Acanthamoeba polyphaga* were among the identified, following comparison with the GenBank results from NCBI (Table 4).

**Table 4:** *Acanthamoeba* T-genotype groups isolated in this study and associated diseases

T-genotype	Species Name	Associated human disease
T1	*Acanthamoeba* spp.^a^	Encephalitis [8, 9]
T2	*Acanthamoeba* spp.^a^, *A. palestinensis, A. pustulosa*	Keratitis and sinusitis [8, 10]
T3	*A. griffini, A. pearcei, Acanthamoeba* spp.^a^,	Keratitis [8]
T4	*A. castellanii, A. polyphaga* ^a^, *A. lugdunensis*, *A. rhysodes*, *A. divionensis*, *A. mauritaniensis*	Keratitis [8, 18]
T5	*Acanthamoeba* spp^a^, *A. lenticulata*	Keratitis [8]
T6	*Acanthamoeba hatchetti* ^a^, *A. palestinensis*	Keratitis [8]
T7	*A. astronyxis*	Unknown
T8	*A. tubiashi*	Unknown
T9	*A. healyi*	Unknown
T10	*A. culbertsoni*	Keratitis and encephalitis [8, 9]
T11	*Acanthamoeba hatchetti* ^a^, *A. stevensoni*, *A.quina*	Keratitis and encephalitis [9]
T12	*A. healyi*	Encephalitis [8, 9]
T13	*Acanthamoeba* spp.	Unknown
T14	*Acanthamoeba* spp.	Unknown
T15	*A. jacobsi*	Keratitis [8]
T16	*Acanthamoeba* spp.	Unknown
T17	*Acanthamoeba* spp.	Unknown
T18	*Acanthamoeba* spp.	Unknown

^a^Isolated in the present study

## Discussion

Over the past decade, *Acanthamoeba* has been the most studied FLA due to its ability to cause and exacerbate disease in humans and animal [3]. Disease effects associated with *Acanthamoeba* often go undetected in Uganda because very little is known about their existence and pathogenic effects, as most communities are completely oblivious of the diseases they might encounter by using contaminated water for cooking, drinking, washing or bathing. The most commonly diagnosed waterborne diseases in Uganda are cholera, campylobacteriosis, shigellosis, salmonellosis and typhoid [16]. Waterborne protozoan parasites *Cryptosporidium* and *Giardia* have also been reported but mostly in wild and domestic animals [17], with very scanty literature available on household water systems. There is no information on diseases caused by amoebae in Uganda and yet FLA of the genus *Acanthamoeba* if present in lethal threshold concentration are highly pathogenic. *Acanthamoeba* has mostly been associated with granulomatous amoebic encephalitis (GAE), amoebic keratitis (AK), cutaneous lesions and sinusitis in humans [8–10]. Eight species of *Acanthamoeba* (*A. castellanii, A. polyphaga, A. culbertsoni, A. hatchetti, A. rhysodes, A. lugdunensis, A. quina* and *A. griffini*) have been previously documented by researchers as aetiologic agents in *Acanthamoeba* keratitis [18] which is common in individuals using dirty contact lenses as well as those washing and bathing with contaminated water [3]. The effects of *Acanthamoeba* infection has been found to be more severe in HIV/AIDS patients and other immunocompromised individuals [8, 9].

The present study investigated the prevalence and molecular genotyping of *Acanthamoeba* in environmental water bodies and communal piped tap water systems in Queen Elizabeth Protected Area, Western Uganda. *Acanthamoeba* prevalence in environmental water was 33 % and in tap water 42.9 %. The low prevalence in environmental samples could be due to a larger number of samples (324) compared to the smaller number of tap water samples (84) collected. The higher prevalence in tap water could also be due to the formation of bacterial biofilms along the water pipes and tap outlet that attracts more predatory *Acanthamoeba* spp. *Acanthamoeba* and bacteria are constantly involved in a predator-prey relationship, with *Acanthamoeba* engulfing and feeding on numerous bacterial colonies [3, 9, 19]. These findings are in agreement with previous studies that detected 51 % and 34 % prevalence in household tap water, all higher than the prevalence in environmental water [20, 21]. Previous studies have also indicated that the prevalence of *Acanthamoeba* in environmental and domestic water can vary from as low as 1 % to as high as 79 % [12, 19, 22–25], all depending on sample size and site.

Taking into consideration the prevalence from a specific environmental sample sources (R. Kyambura (39.6 %), KCB (60.7 %), FLS (50 %) and KMC (5.3 %), it is evident that water from the mid-channel had the least prevalence of the organisms; probably because this water was clearer and less contaminated with organic matter compared to that from other sites. The present study reports high microbial loads of *Acanthamoeba* at KCB (3.44 ± 0.49) and R. Kyambura (3.08 ± 0.53). These same sampling areas were associated with lower levels of DO (mg/l), elevated Temperature (°C) and conductance (μS/m) and TDS (g/l) (Table 2) compared to other sites. We report a significant correlation of *Acanthamoeba* with mean dissolved oxygen (*r* = -.231^**^) and temperature (*r* = -.051). The high prevalence and mean of the organisms at the banks and FLS could be due to contamination of the water by formation of more organic matter from rotting leaves, household refuse and faecal matter dumped by communities living nearby. This increases the build-up of bacteria, consequently increasing the number of *Acanthamoeba* spp. and other amoebae. This concurs with previous findings, that there are more amoebae when there is a build-up of more organic matter in soil and water because the organic fraction contains organic molecules needed for microbial development [26–28]. *Acanthamoeba* spp. are common pathogenic FLA whose distribution is variable and often but not always influenced by physico-chemical parameters [29]. *Acanthamoeba* spp. have been found to be more prevalent in contaminated, bacteria-rich water [29] irrespective of the physico-chemical parameters of the water. However, very high and very low temperature, as well as dissolved oxygen will ultimately lead to low *Acanthamoeba* trophozoite counts. *Acanthamoeba* spp. being ubiquitous, exist in many natural environments and domestic water systems but their numbers fluctuate to a certain extent with precipitating factors like dissolved oxygen, temperature, organic matter, conductivity, oxidation reduction potential, although not so significantly [29].

Classifying the genus *Acanthamoeba* remains a debatable topic but the molecular genotyping being used currently has proved precise in isolate identification, producing more than 25 species. *Acanthamoeba* sequence types have been previously grouped from T1 to T18. In the present study six distinct partial *Acanthamoeba* sequences belonging to sequence T-genotype groups T1, T2, T4, T5, T6 and T11, with only two identical (*Acanthamoeba hatchetti* and *Acanthamoeba polyphaga*) to already known *Acanthamoeba* spp. sequences from NCBI comparisons were obtained. The remaining four *Acanthamoeba* spp. which were not classified to species level may be unique to Uganda. Two isolates belonged to T4, whereas T1, T2, T5, T6 and T11 had only one isolate each. The *Acanthamoeba* strain identified as genotype T4 and T6 was isolated from both environmental and tap water samples. All the other strains were from environmental samples. Previous studies indicate that T2 and T6 are largely environmental isolates that are phylogenetically close to one another [30] although they have also been both isolated from clinical AK cases [31]. *Acanthamoeba* T3 and T11 are closely related to T4 and have been found to also be responsible for AK [32]. *Acanthamoeba* of T4 is reported as the most commonly encountered and most diverse T-genotype group found in both environmental and clinical samples. [30]. Genotype T5 is the second most prevalent [12], mainly isolated from sewage dump locations and AK infections [30].

The findings from the present study indicate that there is a high likelihood of contamination of both environmental and domestic tap water systems with *Acanthamoeba* spp. indicating poor water source quality and possible predisposition of the end users to a variety of infections. Since there are no previous studies on *Acanthamoeba* or any other amoeba in Uganda, it is hard to know the current situation in terms of the effect on human health but we can state that the various genotypes isolated from water samples in the present study could be responsible for silent illnesses that often lead to severe morbidities and mortalities among communities. *Acanthamoeba* genotypes T1 (responsible for encephalitis), T2 (keratitis and sinusitis), T3/T5 (keratitis) and T11 (keratitis and encephalitis) isolated from the water samples pose a high risk to the households in QEPA using both environmental and piped tap water. It is, therefore, imperative that more research on *Acanthamoeba* spp. and other amoebae is done in Uganda to build up knowledge of the types, prevalence, diagnosis, prevention and treatment measures.

## Conclusion

There was a high prevalence of *Acanthamoeba* in communal piped tap and environmental water used by communities (at fish landing sites, channel and river banks). Increasing prevalence of *Acanthamoeba* in water means that there is increased density of prey organisms (bacteria). This can be an indicator of poor environmental and domestic tap water quality.

## Abbreviations

+ve: positive
C: celsius
AK: amoebic keratitis
CNS: central nervous system
FLA: free living amoeba
FLS: fish landing site
*g*: *gravitational force*
g: grams
HIV/AIDS: Human Immunodeficiency Virus/Acquired Immune Deficiency Syndrome
KCB: Kazinga channel bank
KMC: Kazinga mid channel
l: liter
mg: milligrams
ml: milliliters
mM: millimolar
mV: millivolts
NCBI: National Center for Biotechnology Information
No.: number
pg: picogram
pmol: picomoles
Prev: prevalence
QEPA: Queen Elizabeth Protected Area
rpm: revolutions per minute
V: volts
μS: microseconds
